# Uranium Isotopes: From Geosciences to Biomedical Sciences

**DOI:** 10.1007/s40572-026-00564-4

**Published:** 2026-10-08

**Authors:** Anirban Basu, Arijeet Mitra, Catherine M. Lucey, Maia Kelly, Ana Navas-Acien, Kathrin Schilling

**Affiliations:** Department of Environmental Health Sciences, Mailman School of Public Health, Columbia University, New York, NY 10032 USA

**Keywords:** Uranium isotope fractionation, Exposure biomarkers, Source attribution, Environmental biomonitoring, Multi-collector ICP-MS

## Abstract

**Purpose of Review:**

Current biomarkers of uranium (U) exposure do not distinguish among exposure sources or provide information on uranium accumulation within target organs. This review evaluates two complementary uranium isotope systems developed in geosciences and examines their potential as biomarkers of uranium exposure and biological processing.

**Recent Findings:**

Advances in multi-collector ICP-MS have established δ^238^U and uranium activity ratios or (^234^U/^238^U) as independent isotope tracers. Experimental studies show that δ^238^U fractionates during biological uptake, transport, and tissue retention, whereas uranium activity ratios remain largely unchanged after absorption and generally preserve environmental source signatures across groundwater, food webs, and biological systems.

**Summary:**

These complementary isotope systems provide information on both the origin of uranium exposure and its biological fate that cannot be obtained from concentration measurements alone. Although additional experimental validation and human studies are needed, existing analytical capabilities and mechanistic evidence support evaluating two uranium isotope tracers, δ^238^U and the activity ratio, as novel biomarkers of uranium exposure and biotransformation.

**Graphical Abstract:**

**Figure:**
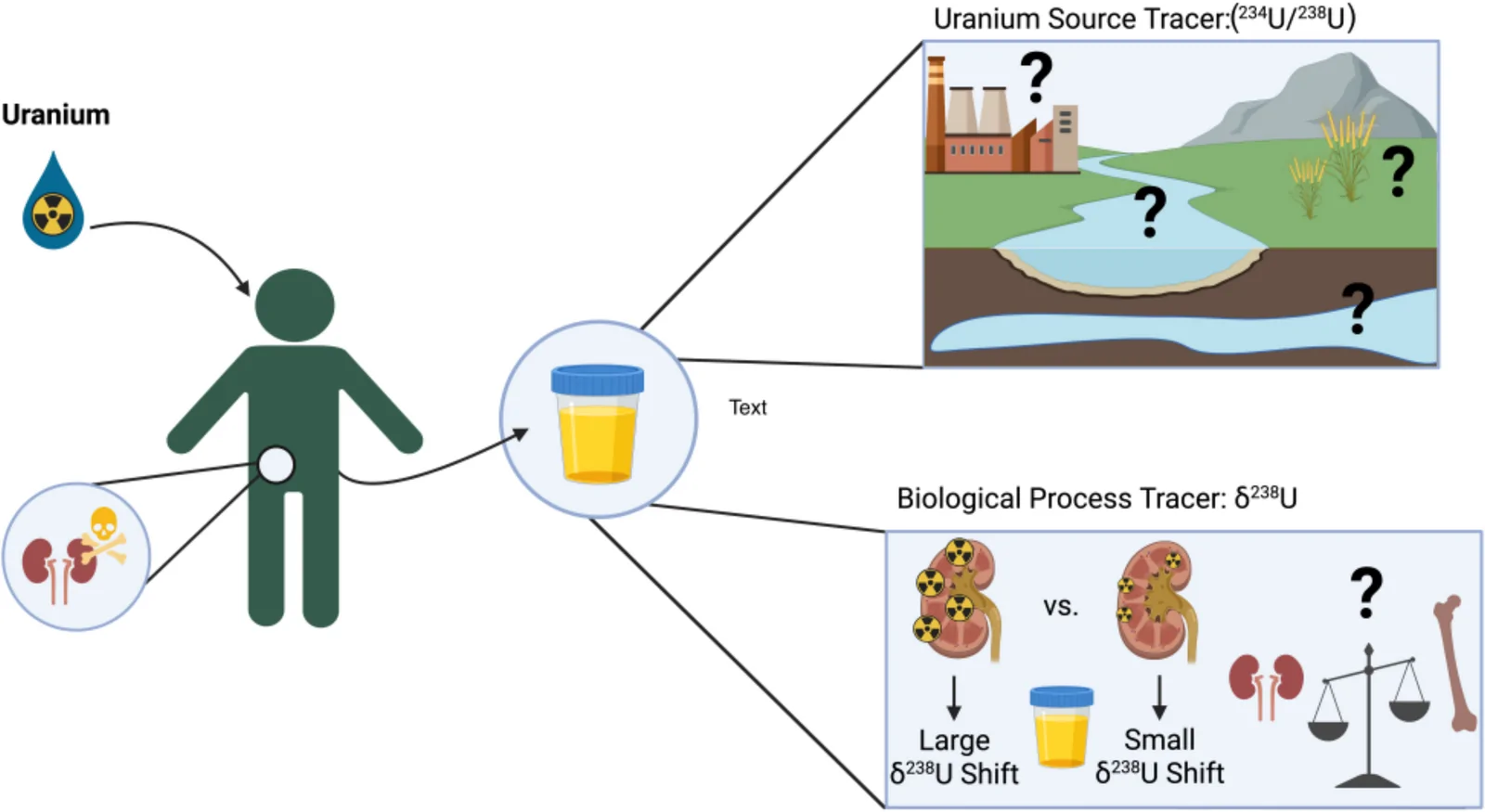

**Supplementary Information:**

The online version contains supplementary material available at https://doi.org/10.1007/s40572-026-00564-4.

## Introduction

Uranium (U) is a ubiquitous naturally occurring element with a long history of industrial, military, and nuclear-energy use. It is among the 50 most abundant elements in Earth’s upper continental crust [1]. It is more abundant than gold, silver and tin, than biologically essential trace elements such as bromine, iodine, molybdenum, and selenium, and even compared to elements with considerable public health concerns, such as cadmium, mercury and radon. Since its discovery in 1789, uranium has been used in industrial processes mainly as a ceramic glaze; since the mid-20th century, however, it is primarily used for weapons and nuclear fuel production [2]. Uranium production for energy has steadily grown over the last decades, and nuclear power currently accounts for about 10% of global power generation. [3]

Global efforts to expand nuclear energy are expected to substantially increase demand for. At the Conference of Parties 28 (COP28) United Nations Climate Conference, more than 20 countries, including the United States (U.S.), pledged to triple nuclear energy capacity by 2050, and six additional countries joined this pledge at COP29 [3]. Since 2020, uranium prices have more than doubled, prompting the reopening of dormant mines like the Pinyon Plain mine in Arizona [4–6]. Global uranium demand is projected to increase from approximately 59,000 metric tons per year in 2024 to between ~ 90,000 and 142,000 metric tons per year by 2050 [7]. This increase implies that uranium production will need to expand substantially, which will increase the risk of its release to the environment. Effective mitigation will require both tracing uranium from environmental sources, including soil, waste, water and food, to human exposure pathways and understanding its uptake, cycling, and health effects in biological systems.

Elevated uranium contamination in drinking water from geogenic sources and mining is widespread globally, including in the United States [8–16]. Chronic uranium exposure in the U.S. general population has only recently gained scientific attention [17, 18]. Drinking-water uranium exposure may be higher for people using unregulated private wells, which serve about 15% of the population, largely in rural areas [18, 19]. About half of domestic wells evaluated contained detectable levels of uranium, and approximately 4% exceeded the Environmental Protection Agency’s maximum contaminant level (MCL) 30 µg/L, compared with about 2% of regulated community water systems. Exposure risk also occurs through these regulated systems, particularly in the Southwest, and uranium was detected in urine from 74% of National Health and Nutrition Examination Survey (NHANES) participants from 2001–2010 [18]. Given this scale of exposure, the associated health implications require careful consideration. Uranium is a biologically non-essential radioactive heavy metal. Although its toxicity is well-established at high concentrations, particularly in the kidneys, bones, liver, reproductive system, nervous system, and lungs, the consequences of long-term, low-level exposure are still poorly characterized [20–25].

Uranium in drinking water is present mainly in its hexavalent form U(VI) [26], a soluble species that enters circulation and produces dose-dependent toxicity [27]. Following ingestion, approximately 1% of U(VI) is absorbed and transported to the kidneys and skeleton, the primary sites of uranium accumulation [28]. Although renal excretion removes most absorbed uranium within 24 h, a fraction is retained long term in target tissues. Chronic low-dose uranium exposure may impair kidney function, and renal injury that remains undetected can lead to irreversible damage. The skeleton serves as the dominant long-term reservoir and contains about two-thirds of the total body burden [28].

Major uncertainties persist over where uranium accumulates in the body during chronic exposure, how long it is retained in different tissues, and whether exposure originates from geogenic sources or legacy mining contamination. The standard biomarker of exposure, urinary uranium concentration, does not capture tissue-specific accumulation or identify the source of exposure [29]. Existing injury markers such as KIM-1 also lack specificity for uranium-induced kidney injury. Uranium isotopic variations may help address these limitations. Well established in geosciences, uranium isotope methods have been widely applied to study geological processes, redox transformations, and uranium cycling in marine systems and modern aquifers [30]. In biomedical sciences, however, their use has been more limited. Because different uranium isotope ratios change predictably during tissue uptake and retention or preserve the fingerprint of the original environmental source, they can provide information on both the biological fate of uranium in the body and the sources of exposure, neither of which can be determined from concentrations alone.

In this review, we examine uranium isotope systematics developed in geosciences and evaluate their potential to trace exposure sources and track uranium cycling in biological systems, including humans and animals. We first introduce the principles underlying the two isotope systems of natural uranium. Then, we evaluate experimental observations, proposed mechanisms, and evidence for δ^238^U (defined below) as a tracer of biological processing and the (^234^U/^238^U) or uranium activity ratio as a tracer of exposure sources.

## Uranium Isotope Systematics: Two Independent Tracers

Natural uranium consists of three isotopes: ^238^U (99.27%), ^235^U (0.72%), and a trace amount of ^234^U (0.005%). Small differences in mass and nuclear properties cause ^238^U and ^235^U to fractionate (i.e., to partition the heavier and lighter isotopes differently between reactants and products) during chemical and biological reactions, shifting their relative abundances between reactants and products [31–33]. These shifts are very small (< 0.1%) but can be resolved by high-precision mass spectrometric measurements, typically at 0.01% precision or better [34–36]. Uranium isotopic composition is expressed as isotope ratios. The delta (δ) notation, reported in per mil (‰), expresses the relative difference in isotope ratios between a sample and a standard, commonly an internationally certified reference material such as CRM 145. The ^238^U/^235^U ratio is expressed as δ^238^U (‰) = [(^238^U/^235^U)_sample_/(^238^U/^235^U)_CRM145_ − 1] × 1000. Negative δ^238^U values indicate enrichment of ^235^U relative to ^238^U. Whereas the ^238^U/^235^U ratio reflects isotopic fractionation during chemical and biological reactions, (^234^U/^238^U) reflects radioactive disequilibrium and represents the ratio of radioactive decay rates [37]. In undisturbed uranium minerals, the activity ratio reaches 1 at secular equilibrium, where the production rate of ^234^U from ^238^U decay equals the decay rate of ^234^U itself. This condition develops over ~ 1 million years [38]. Most natural environmental uranium sources deviate from secular equilibrium and therefore carry distinct activity-ratio signatures [38–41].

Uranium isotope methods have diverse applications in both ancient geological records and modern environmental systems [30]. Early studies, which operated under the assumption that ^238^U/^235^U ratios were invariant, used uranium isotopes to date geological formations and processes, forming the basis of uranium–lead geochronology [42–44]. The development of high-precision multi-collector inductively coupled plasma mass spectrometry (MC-ICP-MS) in the 2000 s overturned the long-standing assumption that ^238^U/^235^U is invariant in nature and established δ^238^U as an entirely new tracer system [45, 46]. More recent work uses δ^238^U to investigate redox-driven uranium cycling in marine environments and modern groundwater systems [30]. Separately, measurements of (^234^U/^238^U) became widely used to trace uranium transport, groundwater residence time, water–rock interaction, and source attribution in aquifers and marine systems [38, 47–51]. These geoscience advances developed the mechanistic framework and analytical precision that now enable uranium isotope applications in biological and biomedical research.

Natural uranium therefore provides two distinct isotope systems that carry orthogonal biological information. The δ^238^U signature records how uranium is processed during biological uptake, transport, and retention because biochemical reactions alter the ^238^U/^235^U ratio during protein-binding and accumulation [52]. In contrast, the uranium activity ratio (^234^U/^238^U) is governed almost exclusively by radioactive decay, and biological reactions do not produce measurable changes beyond current analytical uncertainty (see supporting information). The activity ratio therefore preserves the isotopic signature of the original environmental exposure source.

## δ^238^U as a Tracer of Uranium Processing in Biological Systems

### Fundamental Mechanisms of Uranium Isotope Fractionation

Theoretical work on isotope fractionation shows that, for heavy elements such as uranium, small differences in nuclear size and shape cause isotopes to behave differently during chemical reactions. This phenomenon is known as the nuclear field shift (NFS) or nuclear volume effect (NVE) [31–33, 53]. Bigeleisen [31, 32] developed the theoretical framework and showed that NFS dominates uranium isotope fractionation during U(IV)-U(VI) exchange reactions and explains why ^238^U is anomalously enriched in U(IV) instead of U(VI). This fractionation is termed anomalous because mass-dependent equilibrium effects generally favor the heavier isotope in the more strongly bonded or more oxidized species, whereas NFS reverses this expectation. NFS effects are significant only in high proton-number elements, where the nucleus occupies enough space to influence electron orbital energies. Electron binding energy depends on nuclear volume, nuclear charge radius and the electron density at the nucleus [33]. Larger ^238^U nuclei have more diffuse charge distributions, which weakens electron binding, especially for orbitals with high electron density near the nucleus, such as *s*-orbitals. Interactions among *p*-, *d*-, and *f-*electrons further alter electron density at the nucleus through electron screening effects [33]. As a result, ^238^U preferentially partitions into uranium species with lower electron density at the nucleus, while ^235^U becomes relatively enriched in species with higher electron density, including oxidized U(VI) complexes.

The NFS strongly influences isotope fractionation during uranium reduction [33–36]. Oxidized U(VI) has the electron configuration [Rn], whereas reduced U(IV) has [Rn]5f2. These additional *f*-electrons in U(IV) reduce electron density at the nucleus. Consequently, at equilibrium between U(VI) and U(IV), the larger ^238^U isotope preferentially partitions into reduced U(IV), whereas ^235^U preferentially partitions into oxidized U(VI). Uranium isotope fractionation has been extensively studied during anaerobic reduction of soluble U(VI) to insoluble U(IV) by metal-reducing bacteria such as *Geobacter* and *Shewanella*. [35, 54, 55] However, uranium retains its U(VI) oxidation state following ingestion in mammals, including mice and humans, which rules out a redox fractionation mechanism in vivo. Furthermore, microbial reduction is anaerobic, whereas hypoxic or anoxic conditions are abnormal in mammalian cells.

NFS can fractionate uranium isotopes during both redox reactions and non-redox ligand exchange, including reactions in which uranium remains U(VI), such as uptake in mammals. In aqueous environments, U(VI) primarily exists as uranyl ions (UO_2_^2+^) coordinated with ligands such as carbonate along the equatorial plane (i.e., perpendicular to the linear O = U = O axis) [56]. Relativistic quantum-chemical calculations show that uranium isotope fractionation can occur during U(VI)–U(VI) ligand exchange without altering the central uranyl structure, or oxidation state [57, 58]. Here, ^235^U preferentially partitions into complexes bearing stronger equatorial ligands through ligand exchange reactions during U(VI) binding to proteins and intracellular binding sites [57, 58]. This preferential uptake of ^235^U leaves the remaining unbound uranium relatively enriched in ^238^U, producing measurable shifts in δ^238^U that can trace uptake and retention at cellular and organ levels.

### Uranium Biokinetics and Limitations of Current Biomarkers

Gastrointestinal absorption of ingested uranium is very limited, with approximately 99% eliminated in feces and about 1% absorbed into the bloodstream, based on animal and human data and ICRP biokinetic modeling [28, 59–64]. Circulating proteins like transferrin and ferritin [65] transport absorbed uranium to tissues throughout the body. Of the uranium absorbed into the bloodstream, up to 75% is eliminated in urine within 24 h, while the retained fraction is distributed mainly to the kidneys and skeleton [28, 66–68]. In the kidney, uranium circulates as small complexes that are filtered at the glomerulus and partly reabsorbed in the proximal tubule, resulting in cortical enrichment [69]. This process is also well known for other heavy metals that accumulate in the renal cortex such as cadmium [70]. Renal uranium toxicity primarily affects the proximal tubule, where uranium accumulation can impair tubular reabsorption and damage renal structures, with persistent injury potentially progressing to chronic kidney disease [69]. The skeleton serves as the dominant long-term uranium reservoir and contains approximately two-thirds of the total body burden [28]. Uranium distribution within bone is heterogeneous, with enrichment at the periosteal surface, vascular spaces, and trabecular interfaces, particularly near sites of mineral formation and blood supply [22]. These patterns of differential accumulation among tissues and across timescales are precisely the information that current non-invasive biomarkers cannot resolve.

Urinary uranium, the most widely used exposure biomarker, does not reflect accumulation in the kidney or bone, the primary target organs, and provides no information about exposure source. Tissue burden is therefore difficult to assess in living individuals. Other approaches address only part of this gap. Kidney tubule injury biomarkers such as KIM-1 and MCP-1 indicate tubular injury but are not specific to uranium and cannot track gradual tissue accumulation [71]. Direct quantification of organ burden requires invasive bone or kidney sampling and carries clinical risk, while biokinetic models [28] estimate tissue concentrations indirectly and do not account for inter-individual variability. These limitations highlight the need for biomarkers that capture both tissue-specific accumulation and exposure source.

### In Vitro Evidence for Non-Redox Uranium Isotope Fractionation

Experimental results obtained with freshwater plankton [72], microalgae [73], and human cells [74, 75] show preferential incorporation of ^235^U during non-reductive U(VI) uptake. Recent in vitro experiments directly demonstrate non-redox uranium isotope fractionation during cellular uptake [74, 75]. Paredes et al. demonstrated preferential uptake of ^235^U during intracellular U(VI) accumulation in a human neuroblastoma cell line (SH-SY5Y) [74, 75]. They proposed that a high-affinity membrane transport protein competes with extracellular carbonate ligands for UO_2_^2^^+^ and mediates cellular uptake through ligand exchange. Under the NFS mechanism, the stronger protein-bound complex would preferentially incorporate ^235^U. These observations establish a mechanistic basis for uranium isotope fractionation during biological accumulation and show that preferential incorporation of ^235^U into stronger intracellular binding environments shifts ^238^U/^235^U ratios in biological tissues.

### In Vivo Evidence for Organ-Level Uranium Isotope Fractionation

The first in vivo evidence that uranium isotope fractionation occurs at the organ level and is detectable in urine was reported by Lucey et al. [52] using a controlled drinking-water exposure model in mice. Female apoE −/− C57BL/6 J mice received 50 mg/L natural uranium in a synthetic groundwater solution formulated to resemble human drinking-water chemistry. Animals were continuously exposed for 2, 7, or 14 days. Additional “washout” groups received uranium-free water for 2 or 7 days following an initial 14-day exposure. Uranium isotope compositions of kidney, femur, gastrointestinal tract components, urine, and feces were measured relative to the administered drinking water (CRM 145, δ^238^U = 0.00 ± 0.07‰). These measurements were used to test whether organ-level uranium accumulation produces a detectable urine–source isotopic offset and whether that offset changes predictably during washout.

The primary target organs, kidney and femur, showed consistent and statistically significant enrichment of ^235^U relative to drinking water, reflected by negative δ^238^U values across all exposure durations [52]. Median δ^238^U values in kidneys ranged from − 0.22 to − 0.44‰ after 7 and 14 days of exposure, whereas femurs ranged from − 0.12 to − 0.21‰ over the same period. In contrast, gastrointestinal tract components and feces showed δ^238^U values indistinguishable from drinking water within analytical uncertainty, because the large unabsorbed uranium pool retained the exposure-source composition. Measurable shifts appeared only after absorption, in kidney and bone. Urine exhibited higher δ^238^U values than kidney tissue, consistent with preferential excretion of ^238^U and retention of ^235^U in organs and intracellular compartments.

This effect was even more pronounced in the washout experiments. After uranium exposure ceased, δ^238^U values in kidneys and femurs became substantially more negative, reaching median values of − 1.21‰ and − 0.78‰, respectively, after 7 days of washout. This amplification indicates that ^238^U continued to be preferentially eliminated through urine during metabolic turnover of uranium. ^235^U remained in tissues for longer periods, further enriching the isotopically light uranium pool in target organs. These observations are consistent with NFS-driven preferential release of ^238^U from uranium–protein bonds during metabolic turnover and with preferential retention of ^235^U in stronger ligand environments within kidney and bone.

### Proposed Mechanisms of Organ-Level Uranium Isotope Fractionation

**Fig. 1:**
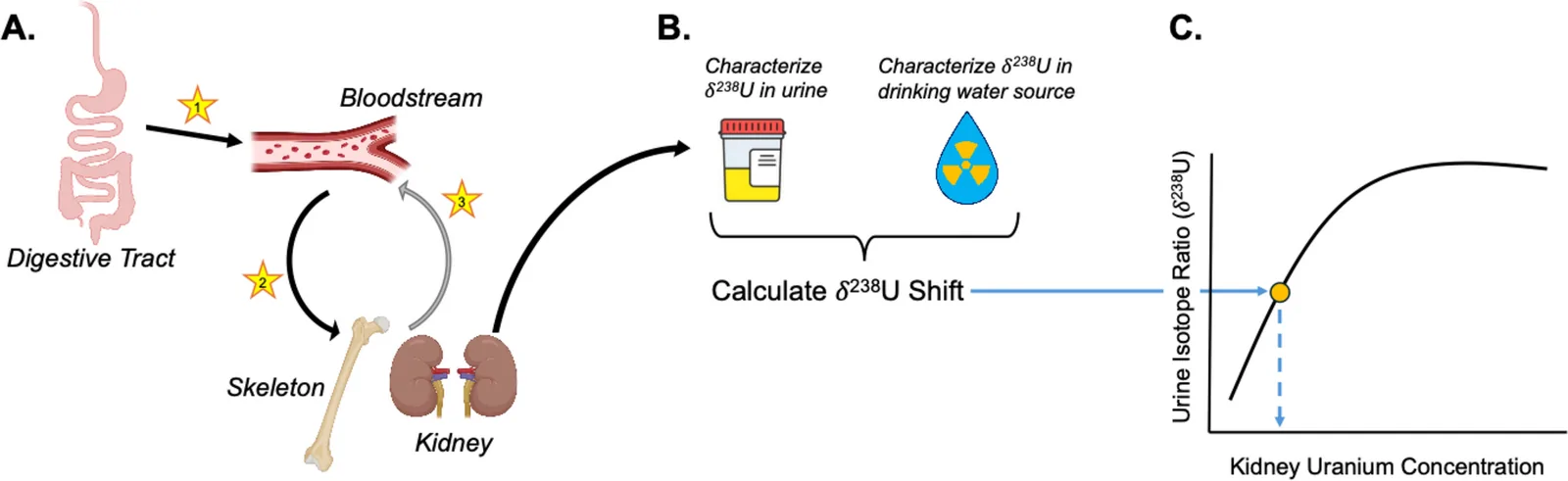
Theoretical framework for using urinary δ^238^U as a tracer of biological processes. **A** Isotopic fractionation of ingested uranium may occur during three biological processes (yellow stars) 1) absorption uranium from the digestive tract into the bloodstream, 2) transfer of uranium from the bloodstream into tissues such as kidneys and bones, and 3) re-distribution of uranium from tissue into the bloodstream. **B** Because uranium released from tissues is recirculated through the bloodstream and eventually excreted in urine, urinary δ^238^U is expected to reflect the isotopic composition of uranium stored in or released from tissues. When the δ^238^U of the exposure source, such as drinking water, is known, the extent of biological uranium processing can be quantified by calculating the difference, or isotopic shift, between urinary δ^238^U and source δ^238^U.** C** The magnitude of the isotopic shift between the source and urine can then be used to estimate the concentration of uranium in a given tissue by mapping the observed shift onto an experimentally defined relationship between isotopic fractionation and tissue uranium concentration. This concentration–fractionation relationship is tissue specific because uranium influx, efflux, and residence times differ among organs

The findings of Lucey et al. [52] introduce δ^238^U as a potential non-invasive urinary tracer of organ-level uranium accumulation, analogous to the use of calcium isotope ratios in urine as biomarkers of bone gain and loss [76–78]. The drinking-water–urine δ^238^U offset is therefore a candidate indicator of organ-level uranium processing, although a quantitative relationship between urinary δ^238^U and kidney or skeletal uranium burden needs to be established through isotope-enabled biokinetic modeling. The mechanistic basis of δ^238^U fractionation that underlies this biomarker potential likely begins during uranium transport in blood and continues during cellular uptake and tissue accumulation (Fig. 1).

In serum, uranyl-carbonate complexes interact with transport proteins involved in uranium delivery to kidney and bone. These interactions indicate that uranium exploits existing metal transport pathways to reach these target organs. U(VI) binds strongly to transferrin and fetuin-A, proteins involved in iron and calcium transport, respectively [56, 65, 79, 80], but has a higher affinity for transferrin than for other serum metalloproteins and undergoes sequential ligand exchange in which carbonate ligands are replaced by tyrosine and aspartate residues [56, 65]. This reaction is thermodynamically favorable and kinetically rapid [56, 65, 80]. Fetuin-A also binds U(VI) strongly (K_d_: 3.00 × 10⁻⁸) and carries more than 80% of serum U(VI) despite its lower abundance relative to albumin, immunoglobulin G, and transferrin [79]. Replacement of carbonate ligands by stronger protein ligands should preferentially enrich and retain ^235^U within protein-bound uranium complexes through NFS-driven isotope fractionation.

Additional fractionation likely occurs after cellular uptake. In the kidney, transferrin receptor 1 mediates uptake of uranyl-loaded transferrin through receptor-mediated endocytosis [81, 82]. Uranium subsequently accumulates within lysosomes as U(VI)-phosphate complexes [83] and, at higher concentrations, as insoluble U(VI)-phosphate precipitates [84]. Phosphate binding is highly favorable under acidic intracellular conditions because U–O bond lengths are shorter in phosphate than carbonate complexes [85, 86]. In bone, uranyl ions exchange with calcium at hydroxyapatite surfaces and accumulate within osteoblasts and osteoclasts, where U(VI) undergoes biomineralization and forms the U(VI)-phosphate mineral meta-autunite [87, 88]. These phosphate interactions provide additional opportunities for preferential enrichment and retention of ^235^U in kidney and bone, while the remaining uranium pool becomes relatively enriched in ^238^U. Fractionation initiated during serum transport may therefore be amplified during intracellular phosphate binding, mineral precipitation, and long-term uranium retention within target organs. Lucey et al. [52] suggested that these processes contribute to the increasing urinary δ^238^U offset observed during washout.

From a translational perspective, Lucey et al. [52] proposed that the offset between drinking water δ^238^U and urinary δ^238^U reflects isotopic partitioning during uranium retention in target organs and therefore provides a potential indicator of uranium accumulation in physiological reservoirs such as the kidneys (Fig. 1). They further demonstrated the feasibility of population-level biomonitoring by showing that 24-h urine collections from moderately exposed populations contain sufficient uranium for isotopic analysis. For example, in the Strong Heart Family Study (SHFS), a median urinary concentration of 0.037 μg/L [89] corresponds to approximately 48 ng of uranium in a 24-h urine collection (1.3 L/day), which is more than three times the amount required for isotopic analysis.

## (^234^U/^238^U) as a Source Tracer of Uranium Exposure

Whereas the ^238^U/^235^U ratio reflects fractionation during biological uranium processing, the second uranium isotope system, the (^234^U/^238^U) or uranium activity ratio, addresses a fundamentally different question: not what happens to uranium after exposure, but where it came from before entering the body.

### Fundamental Mechanisms of (^234^U/^238^U) Disequilibrium

Variations in the (^234^U/^238^U) or uranium activity ratio arise from α-recoil, preferential leaching, and dissolution over geological timescales [37, 39, 40, 90]. During α-decay of ^238^U, emission of an α-particle imparts recoil energy to the daughter nuclide ^234^Th, which can be displaced by several tens of nanometers from the mineral lattice and released into surrounding water during water–rock interaction [39, 90, 91]. The displaced ^234^Th (half-life = 24 days) rapidly decays to ^234^U, which produces the excess ^234^U that characterizes many groundwater systems. Recoil-induced lattice damage further enhances preferential leaching of radiogenic ^234^U during dissolution. Differences in mineralogy, weathering rate, water–rock contact time, redox conditions, and access to recoil-damaged grain surfaces control preferential ^234^U release and thus generate location-specific (^234^U/^238^U). The resulting case-specific activity-ratio signatures in groundwater, surface water, and soils can therefore be used to trace uranium transport and distinguish environmental sources. Measurements of uranium activity ratios have therefore been used to investigate uranium derived from geogenic sources, mining, and industrial discharges [41, 92, 93].

### Biological Invariance of the Activity Ratio

**Fig. 2:**
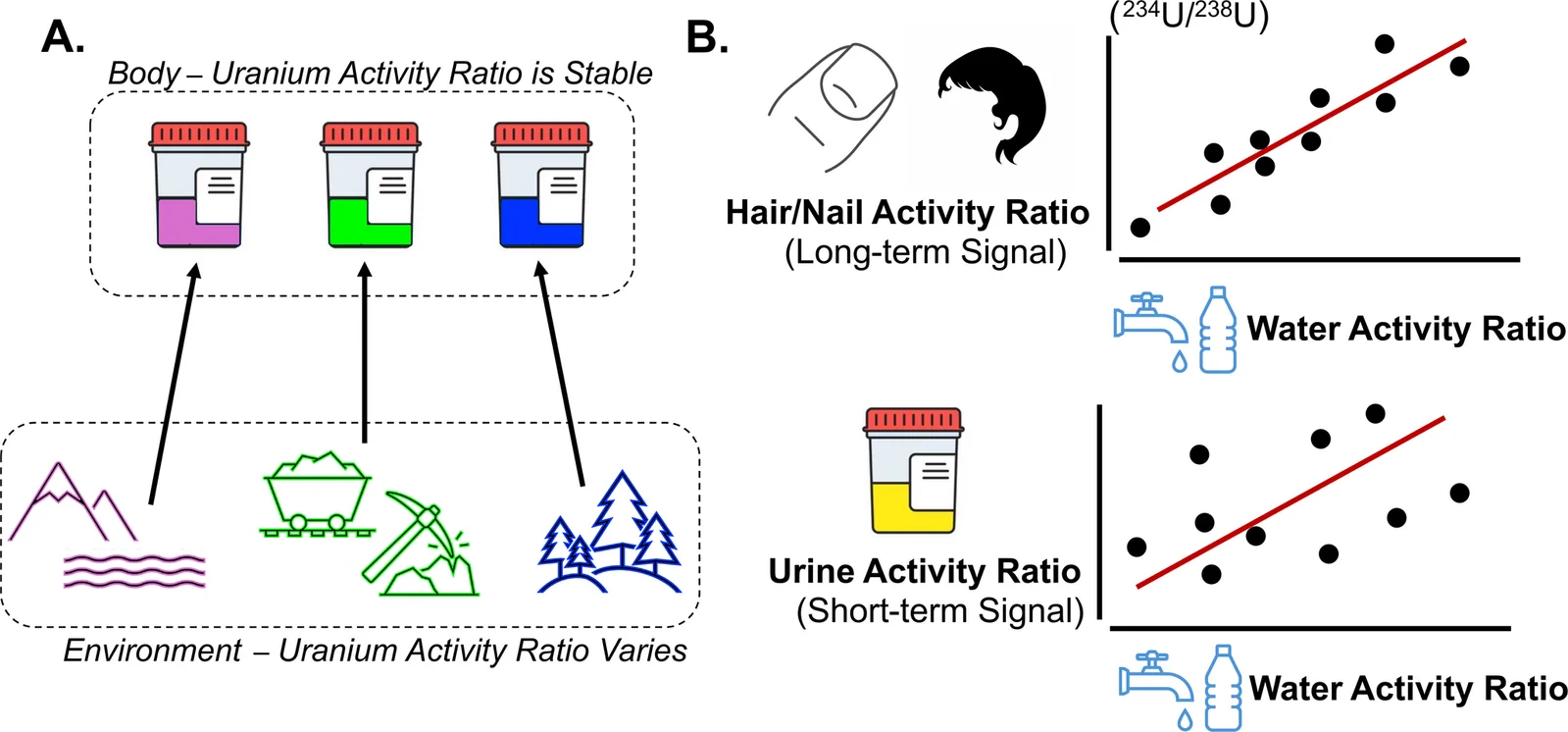
Uranium activity ratios are biomarkers of exposure source. **A** The (^234^U/^238^U) varies among environmental sources as a result of geological and geochemical processes. In contrast, biological uptake and processing are assumed not to measurably fractionate the (^234^U/^238^U). Consequently, the source-specific environmental (^234^U/^238^U) signature is preserved in biological samples and can be used to identify or distinguish the original source of uranium exposure. **B** Hair and nails integrate uranium exposure over longer periods and are therefore expected to provide relatively stable, long-term (^234^U/^238^U) signals that correlate strongly with the (^234^U/^238^U) of exposure sources, such as drinking water. By contrast, urine reflects more recent uranium intake, and its (^234^U/^238^U) may vary over time as exposure sources or their relative contributions change. Urinary (^234^U/^238^U) may therefore remain correlated with drinking-water activity ratios, but the relationship is expected to be weaker than that observed for hair or nails

Activity-ratio signatures acquired from water, food, or inhalation are preserved during transport through the body and can be measured in biological samples. Because the half-lives of ^234^U (~ 245,000 years) [94] and ^238^U (4.5 billion years) [95] greatly exceed physiological residence times of uranium, radioactive decay during biological processing is negligible. Any NFS-driven isotope effects associated with biological processing of uranium are smaller than α-recoil-induced disequilibrium and are not resolvable in the activity ratio. Applying Bigeleisen's [31] NFS scaling (K = 1.194) to the δ^238^U fractionation measured by Lucey et al. [52], the predicted uranium activity ratio shift from biological processing remains 3.5- to 19-fold smaller than the full procedural reproducibility threshold of 0.01 (2 × root mean square error) determined from independently prepared duplicate samples across the evaluated groundwater source activity-ratio range of 1.0–5.4 (see Supporting Information) [96]. Post-exposure washout was evaluated separately because it represents Rayleigh-type distillation of a shrinking, unreplenished tissue uranium reservoir. After bounding the single most fractionated washout measurement at the largest independently calculated non-redox nuclear field-shift magnitude for U(VI)–U(VI) ligand exchange and applying the same conversion [57, 58], the predicted shift remains 1.4-fold smaller than full-procedure reproducibility, even at a source activity ratio of 5.4. These calculations indicate that biological fractionation is not expected to measurably overprint activity-ratio source information during ongoing exposure or under the bounded Rayleigh-type washout scenario evaluated here. The uranium activity ratio therefore retains information about exposure sources even within a biological system (Fig. 2).

### Transmission of Source Signatures through Food Webs

Diet and drinking water are major pathways of uranium exposure. Preservation of environmental (^234^U/^238^U) signatures during biological uptake and trophic transfer is a prerequisite for identifying drinking-water and dietary uranium sources in human biospecimens. Available evidence indicates that these signatures are generally preserved, although some variability has been reported among tissues, species, and environments.

In terrestrial plants, uranium concentrations generally decrease from roots to leaves, stems, and reproductive tissues [97, 98]. Root tissues typically show activity ratios similar to those of surrounding soil porewater and groundwater [99]. Reported activity ratios in above-ground tissues may reflect both internal uptake and surface deposition. One study reported higher activity ratios in leaves and stems than in roots [98], a pattern that could arise from preferential root-to-shoot transfer of ^234^U or surface deposition of resuspended soil and dust on aerial tissues. In cultivated leafy vegetables with rough or hairy surfaces (carrot, parsley, lettuce), activity ratios are essentially uniform across roots and aerial tissues (0.96–0.99), even though uranium concentrations are markedly higher above ground [100]. Therefore, current evidence supports preservation of environmental source signatures during plant uptake and translocation, although reports of activity-ratio variability require independent verification.

Animal studies similarly indicate that (^234^U/^238^U) activity ratios generally preserve environmental source signatures. Aquatic organisms provide the clearest evidence because they remain continuously immersed in a single uranium source. Phytoplankton and zooplankton from the southern Baltic show activity ratios of 1.13–1.16, matching Baltic and oceanic water values of 1.14–1.17 [101–103]. Antarctic marine biota (algae, fish, molluscs, mammals) have an average activity ratio of 1.12 [104], consistent with global ocean water (1.15) [104]. Fish from Kurday Pit Lake generally show activity ratios close to 1, in agreement with the local water ratio of 1.03 ± 0.03 [105]. These close matches indicate that environmental source signatures are preserved under stable, single-source exposure.

Interpretation is more complex in terrestrial and free-ranging animals because dietary sources are rarely characterized and tissues integrate exposure over different timescales. In marine birds from the southern Baltic, activity ratios range from 0.35 in muscle to 1.25 in skeleton [106]. Several of the sampled species are migratory, so skeletal activity ratios may integrate exposure across an entire migratory range, whereas kidney activity ratios may primarily reflect recent local intake. A similar limitation applies to wild deer, where activity ratios range from 0.61 to 1.42 across kidney, liver, and muscle [107], but bone was not sampled and seasonal foraging ranges were not characterized.

The most controlled organ-level study examined a juvenile domestic goat raised on a constant feed source with a uranium activity ratio of 1.25 ± 0.07 for 39 days [108]. After exposure, activity ratios were 1.4 ± 0.4 in kidney, 1.6 ± 0.3 in liver, 1.5 ± 0.2 in lung, 1.7 ± 0.3 in bladder, 1.3 ± 0.17 in bone, and 1.3 ± 0.3 in small intestine. Within the reported uncertainties, most individual organ measurements cannot be distinguished confidently from the feed ratio, and evidence for organ-specific differences rests primarily on the pooled soft-tissue estimate (1.57 ± 0.13). However, this pooled value has little physiological meaning because it averages organs with different uranium kinetics. According to the authors, soft tissues acquire isotopically fractionated uranium from circulating blood, whereas bone and gut wall retain the original feed ratio. This interpretation implies that uranium in circulating blood becomes isotopically modified. If so, bone should incorporate that altered uranium pool during mineralization rather than preserve the original feed ratio. Prenatal uranium was not completely replaced during the experiment. If the activity ratio of the prenatal uranium, which was not measured, differed from that of the feed, it may have contributed to the organ activity ratios measured at sacrifice. The differing isotope signatures among bone, soft tissues, and urine likely reflect distinct integration periods: bone can retain a longer-term exposure record, whereas soft tissues and urine are more responsive to recent uptake, turnover, and elimination.

Overall, uranium activity ratios generally preserve source signatures during transfer through food webs and can therefore trace environmental sources in human biomonitoring.

### Human Biomonitoring Applications of (^234^U/^238^U)

Hair and nails show the closest correspondence with drinking-water activity ratios. In a subgroup of 45 Finnish private well users selected from a cohort of 205 individuals and exposed to drinking-water uranium concentrations of 0.13–1500 µg/L, hair and toenail activity ratios closely matched those of drinking water (R^2^ = 0.97), whereas the water–urine relationship was weaker (R^2^ = 0.72) [109, 110]. Similarly, in Swedish well-water users, the water–hair correlation (r^2^ = 0.91) substantially exceeded the water–urine correlation (r^2^ = 0.56) [111]. Weaker water–urine correlations indicate greater sensitivity to recent intake, mixed sources, and release from retained uranium pools. Nevertheless, urinary activity ratios may help with source attribution when the dominant sources are characterized and isotopically distinct. Keratinous tissues such as hair and nails integrate uranium exposure over weeks to months, whereas urine primarily reflects intake over days [109, 111–113]. Hair therefore provides a more stable record of chronic exposure, whereas urinary activity ratios are more sensitive to short-term variation in dietary and drinking-water sources (Fig. 2B). Recent ICP-MS methods have further improved analytical precision and detection limits, particularly for urine [114–116], but systematic cross-tissue activity-ratio measurements in populations exposed to natural uranium remain unavailable.

## Integrating Dual Uranium Isotope Tracers

Urine is a noninvasive matrix in which simultaneous measurement of uranium concentration, (^234^U/^238^U), and δ^238^U could add source and biological-processing information to conventional exposure assessment. These tracers provide orthogonal information: the activity ratio or (^234^U/^238^U) preserves information about exposure sources, whereas δ^238^U reflects biological processing of uranium after uptake. This combined framework addresses two complementary questions: where uranium originated and how it was processed after absorption.

The Strong Heart Water Study region in the Northern Plains illustrates the potential of this framework. Mitra et al. [10] constructed the first simultaneous δ^238^U and activity ratio groundwater isoscapes (spatial maps of isotope ratios across a geographic region) for sandstone aquifers beneath tribal lands in South Dakota, where residents have significant, well-characterized uranium exposure. The isoscapes showed that groundwater from the oxidizing northeastern part of the Tribal land contained 23–48 μg/L uranium and had activity ratios of 1.53–1.80, whereas groundwater from the reducing southwestern part exceeded 1.83. A participant with a urinary activity ratio matching one of these groundwater fingerprints could therefore be linked to a specific exposure source, although this information is not yet available. Simultaneous measurement of urinary δ^238^U could then identify biological processing of uranium, potentially before other biomarkers of uranium-related toxicity such as KIM-1, MCP-1, or standard kidney function markers become abnormal.

Both the δ^238^U and activity-ratio frameworks can now be evaluated in population studies using existing MC-ICP-MS techniques. The findings of Lucey et al. [52] establish the feasibility of implementing both isotope tracers in population level biomonitoring. The dual-isotope framework is ready for near-term implementation because groundwater isoscapes already provide spatially resolved source signatures. A corresponding urinary isoscape would quantify how biological uptake, retention, and excretion modify those signatures as uranium passes through the body. It would provide the first population-scale map of human uranium bioavailability. The Strong Heart Family Study provides an immediately actionable platform for this work as large urine volume samples can be obtained from participants in the same regions where water uranium isoscapes are also available [10]. Baseline urinary uranium concentrations are already available, so isotopic tracing can proceed without a separate analytical feasibility or exposure-characterization phase. Two scientific requirements must be addressed before this framework can be fully translated to population studies. First, a uranium isotope biokinetic model is needed to describe how δ^238^U evolves through blood, kidney, and bone compartments and to predict urinary δ^238^U as a function of organ uranium burden. The ICRP biokinetic model provides the physiological foundation but would require extension to include isotope-ratio evolution through fractionation at compartment boundaries [28]. Second, concurrent drinking-water, dietary, and urine sampling is needed to determine how environmental source signatures are transferred to human biospecimens and how urinary isotope signals evolve with exposure duration and intervention. The δ^238^U evidence currently derives from a limited number of experimental studies, and blood uranium concentrations may be too low for routine isotope-ratio measurements [117–119]. In the mouse experiment, for example, blood uranium concentrations remained near 1 ng/g throughout exposure despite progressive organ accumulation [52]. At environmental exposure levels, blood is therefore not a practical matrix for δ^238^U measurements. These limitations do not challenge the underlying dual-isotope framework. Instead, they define the critical next steps needed to translate uranium isotope biomarkers into population-based studies.

## Conclusions

Current biomarkers, particularly urinary uranium concentrations, do not distinguish among environmental exposure sources and provide limited information on uranium accumulation within target organs. Two complementary uranium isotope systems can address these limitations by providing information that concentration measurements alone cannot. Originally developed to investigate geological processes, redox transformations, and uranium cycling in natural systems, these isotope methods now provide the mechanistic and analytical foundation for biological and biomedical applications. Activity ratios preserve environmental source signatures across groundwater, food webs, and human biospecimens, whereas δ^238^U reflects biological processing after absorption and may provide information on uranium accumulation and retention within target tissues.

The evidence reviewed here shows that δ^238^U shifts during non-redox biological uptake, transport, and retention of uranium, with the strongest experimental evidence coming from cell studies and a controlled mouse exposure model. In that model, kidney and bone became enriched in ^235^U relative to drinking water, and urinary δ^238^U changed predictably during washout, linking the urine signal to organ-level uranium retention. In contrast, activity ratios remain effectively invariant over physiological timescales and generally preserve environmental source signatures during transfer through groundwater, plants, animals, and human biospecimens. Remaining uncertainties include the limited number of δ^238^U experimental studies and the need to verify whether reported activity-ratio variability reflects biological processing or differences in source signatures and uranium bioavailability.

The application of δ^238^U and activity ratio measurements extend the application of uranium isotope methods from geosciences to environmental health. Continued development of biokinetic models, controlled experimental studies, and well-characterized human populations will determine the utility of these tracers for biomonitoring and environmental health research. If successful, dual uranium isotope tracers could establish a new approach for investigating uranium exposure, tissue accumulation, and toxicity in human populations.

## Supplementary Information

Below is the link to the electronic supplementary material.

Supplementary file1 (DOCX 26 KB)

## Data Availability

No datasets were generated or analysed during the current study.
