# Supplementary material for: Uranium Isotopes: From Geosciences to Biomedical Sciences
Source: Curr Environ Health Rep. 2026 Oct 8;13(1):44. doi: 10.1007/s40572-026-00564-4 (PMC13649861; doi:10.1007/s40572-026-00564-4)
Supplement: Supplementary file 1 [file 40572_2026_564_MOESM1_ESM.docx]

Supporting Information

Uranium Isotopes: from geosciences to biomedical sciences

Anirban Basu^1^, Arijeet Mitra^1^, Catherine M. Lucey^1^, Maia Kelly^1^, Ana Navas-Acien^1^, Kathrin Schilling^1^

Department of Environmental Health Sciences, Mailman School of Public Health, Columbia University, New York, NY, 10032, United States

**1.** **Biological Uranium Isotope Fractionation and Activity-Ratio Reproducibility**

The nuclear field shift (NFS) scaling factor, K = 1.194, is the ratio of the NFS magnitude for the ^234^U–^238^U isotope pair to that for the ^235^U–^238^U pair. We derived K from the NFS data reported by Bigeleisen.^1^ Because measured δ^238^U quantifies the fractionation of ^238^U relative to ^235^U, we used K to predict the corresponding per mil change in ^234^U/^238^U:

δ(^234^U/^238^U) = −K × δ^238^U	(1)

We then converted this value to an absolute change in the activity ratio:

ΔAR = AR_source_ × δ(^234^U/^238^U)/1000	(2).

Here, ΔAR is the difference between the activity ratio after biological processing and the source activity ratio and therefore quantifies how much biological fractionation could alter the source signature. Equation 1 scales the measured ^238^U/^235^U fractionation to the corresponding predicted fractionation in ^234^U/^238^U. Equation 2 converts this predicted per mil change into an absolute activity-ratio shift at the source activity ratio evaluated.

**Table 1. Predicted effects of biological U isotope fractionation on the ^234^U/^238^U activity ratio.** Calculations apply the NFS scaling factor, K = 1.194, using Equations 1 and 2. Predicted absolute activity-ratio shifts are compared with the 0.01 full-procedure reproducibility threshold (2 × root mean square error or RMSE).

| **Condition** | **δ^238^U input (‰)** | **AR_source** | **Predicted δ(^234^U/^238^U) (‰)** | **Predicted \|ΔAR\|** | **Relative to 0.01 threshold** |
| --- | --- | --- | --- | --- | --- |
| Ongoing exposure: maximum observed | −0.44 | 1.0 | +0.525 | 0.0005 | 19.03-fold below |
| Ongoing exposure: maximum observed | −0.44 | 5.4 | +0.525 | 0.0028 | 3.52-fold below |
| Washout: raw extreme, shown for comparison | −2.04 | 5.4 | +2.436 | 0.0132 | 1.32-fold above |
| Washout: bounded non-redox maximum | −1.14 | 5.4 | +1.361 | 0.0074 | 1.36-fold below |
| Washout: carbonate special case | −0.67 | 5.4 | +0.800 | 0.0043 | 2.31-fold below |

*Worked limiting cases:* For ongoing exposure at AR_source_ = 5.4, Equation 2 gives 5.4 × (1.194 × 0.44)/1000 = 0.0028, which is 0.01/0.0028 = 3.52-fold below the reproducibility threshold. For the bounded washout case, Equation 2 gives 5.4 × (1.194 × 1.14)/1000 = 0.0074, which is 0.01/0.0074 = 1.36-fold below the threshold.

**Treatment of Post-Exposure Washout**

In the experiments by Lucey et al.,^2^ ongoing exposure continuously replenished tissue uranium and therefore provided the appropriate basis for estimating the activity-ratio signatures recorded by organs and other physiological reservoirs. In contrast, post-exposure washout represented a non-steady-state, Rayleigh-type distillation process in which the tissue uranium reservoir shrank without replenishment, while preferential loss of ^238^U progressively enriched the residual pool in ^235^U. Table 1 retains the raw −2.04‰ washout value for transparency. For the washout calculation, however, we bounded the fractionation at 1.14‰, the largest independently calculated non-redox NFS magnitude for the U(VI)–U(VI) ligand-exchange systems evaluated by Sato et al.^3^ We then applied the same scaling factor, K = 1.194, to this bounded value.

**2. Full-Procedure Reproducibility Threshold**

Published estimates (e.g., refs^4–6^) of analytical precision for activity ratios are commonly based on repeated measurements of a standard or a single purified solution while sample preparation is held constant. These estimates may overstate analytical precision and underestimate the uncertainty associated with independently processed samples. So, we compared the predicted activity-ratio shifts with a full-procedure reproducibility threshold of 2 × RMSE = 0.01. We calculated this threshold from independent duplicate analyses in which separate aliquots of the same sample underwent separate column separations and, in several cases, were measured on different days. These duplicate differences capture variability from chemical separation, column-to-column differences, and between-session instrumental drift. The 0.01 threshold represents sample-level, full-procedure reproducibility and not the instrument-level 2σ precision.

**3. Interpretation and Model Assumptions**

These calculations are model-based because biological fractionation of ^234^U/^238^U has not been measured directly. The conversion assumes that NFS dominates the relevant biological isotope fractionation and follows the scaling factor K = 1.194. During ongoing exposure, the estimated activity-ratio shift is 3.5- to 19-fold smaller than the full-procedure reproducibility threshold.

**References**

1.	Bigeleisen J. Nuclear size and shape effects in chemical reactions. Isotope chemistry of the heavy elements. J Am Chem Soc. 1996 Apr 17;118(15):3676–80. doi:DOI 10.1021/ja954076k

2.	Lucey CM, Pearson BL, DeSantis K, Goldsmith J, Halliday AN, Navas-Acien A, et al. Isotopic Fractionation of Natural Uranium in Mice as a Potential Biomarker of Renal Accumulation. Environmental Science & Technology. 2025 Jul 14. doi:10.1021/acs.est.5c03403

3.	Sato A, Hada M, Abe M. Electron correlation effects on uranium isotope fractionation in U(VI)–U(VI) and U(IV)–U(VI) equilibrium isotopic exchange systems. Phys Chem Chem Phys. 2024 May 29;26(21):15301–15. doi:10.1039/D4CP01149J

4.	Cheng H, Lawrence Edwards R, Shen CC, Polyak VJ, Asmerom Y, Woodhead J, et al. Improvements in 230Th dating, 230Th and 234U half-life values, and U–Th isotopic measurements by multi-collector inductively coupled plasma mass spectrometry. Earth and Planetary Science Letters. 2013 Jun 1;371–372:82–91. doi:10.1016/j.epsl.2013.04.006

5.	Hu HM, Shen CC, Cheng H, Woodhead JD, Edwards RL, Zhao J xin, et al. Sub-epsilon natural 234U/238U measurements refine the 234U half-life and U-Th geochronology. Science Advances. 2025 Oct;11(40):eadu8117. doi:10.1126/sciadv.adu8117

6.	Veerasamy N, Takamasa A, Murugan R, Kasar S, Aono T, Inoue K, et al. Chemical Separation of Uranium and Precise Measurement of 234U/238U and 235U/238U Ratios in Soil Samples Using Multi Collector Inductively Coupled Plasma Mass Spectrometry. Molecules. 2020 Jan;25(9):2138. doi:10.3390/molecules25092138
